# Translobar venous drainage management in (sub)lobar resections: Virtual 3-dimensional planning and surgical technique

**DOI:** 10.1016/j.xjtc.2023.03.021

**Published:** 2023-04-17

**Authors:** Wouter Bakhuis, Lea Betser, Amir H. Sadeghi, Dominique Gossot, Denis Susa, Alexander P.W.M. Maat, Sabrina Siregar, Ad J.J.C. Bogers, Agathe Seguin-Givelet, Edris A.F. Mahtab

**Affiliations:** a Department of Cardiothoracic Surgery, Erasmus Medical Center, Rotterdam, The Netherlands; b Surgery Department, Institut Montsouris, Institut du Thorax Curie Montsouris, Paris, France; c Bravis Hospital, Roosendaal, The Netherlands; d Paris 13 University, Sorbonne Paris Cité, Faculty of Medicine SMBH, Bobigny, France

**Figure:**
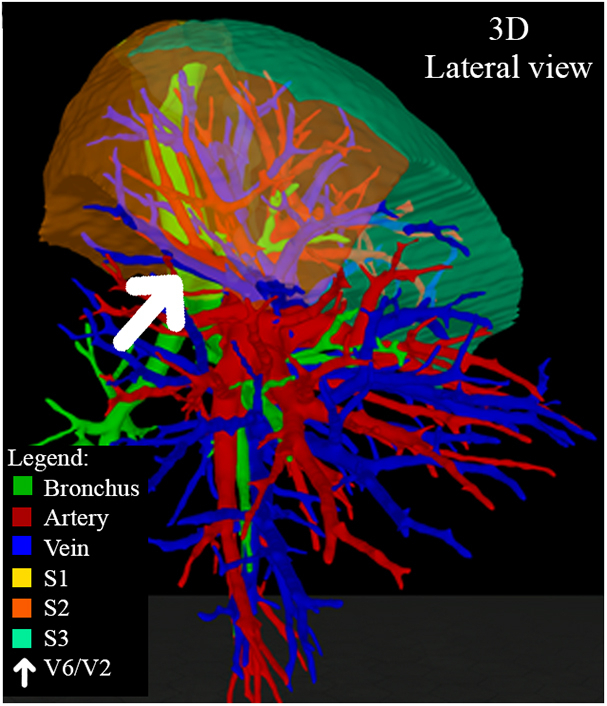
Lateral 3D visualization of right-sided interlobar vein between segment 2 and 6.

**Central Message**

To emphasize the importance of thorough preoperative planning, 5 cases with translobar venous drainage of the right lung, observed during 3-dimensional and virtual reality visualization are described.

Most of the currently available knowledge of bronchovascular anatomy is based on cadaver studies.^1^ However, bronchovascular anatomy is highly variable, advocating the term patient-specific anatomy instead of anatomical variation.^2^ With innovative visualization techniques such as virtual 3-dimensional (3D) modeling and immersive virtual reality (VR), patient-specific anatomy can be visualized with more detail and accuracy than through conventional 2-dimensional computed tomography (2D-CT) images.

In our centers, we have adopted novel 3D/VR technologies (Pulmo3D, Visible Patient, and PulmoVR) to preoperatively analyze patient-specific anatomy for pulmonary resections. Over the past few years, we have faced several cases in which there was a right-sided translobar venous drainage in patients undergoing (sub)lobar resection. In this article, we aim to describe the importance of a thorough preoperative planning strategy and surgical technique to either spare or selectively ligate these translobar veins.

## 3D Modeling Routine, Case Presentation, and Surgical Technique

VR/3D-based planning, using PulmoVR and Pulmo3D, has been used since 2020 in Erasmus Medical Center and Bravis Hospital, and Visible Patient has been used since 2016 in the Institut du Thorax Curie Montsouris for all segmentectomies, as described elsewhere.^3^^,^^4^ Five patient cases with translobar venous drainage of the right lung were identified. Written informed consent was obtained from all patients. The study was approved by the medical ethical committee of the Erasmus Medical Center (MEC-2020-0702).

**Figure 1:**
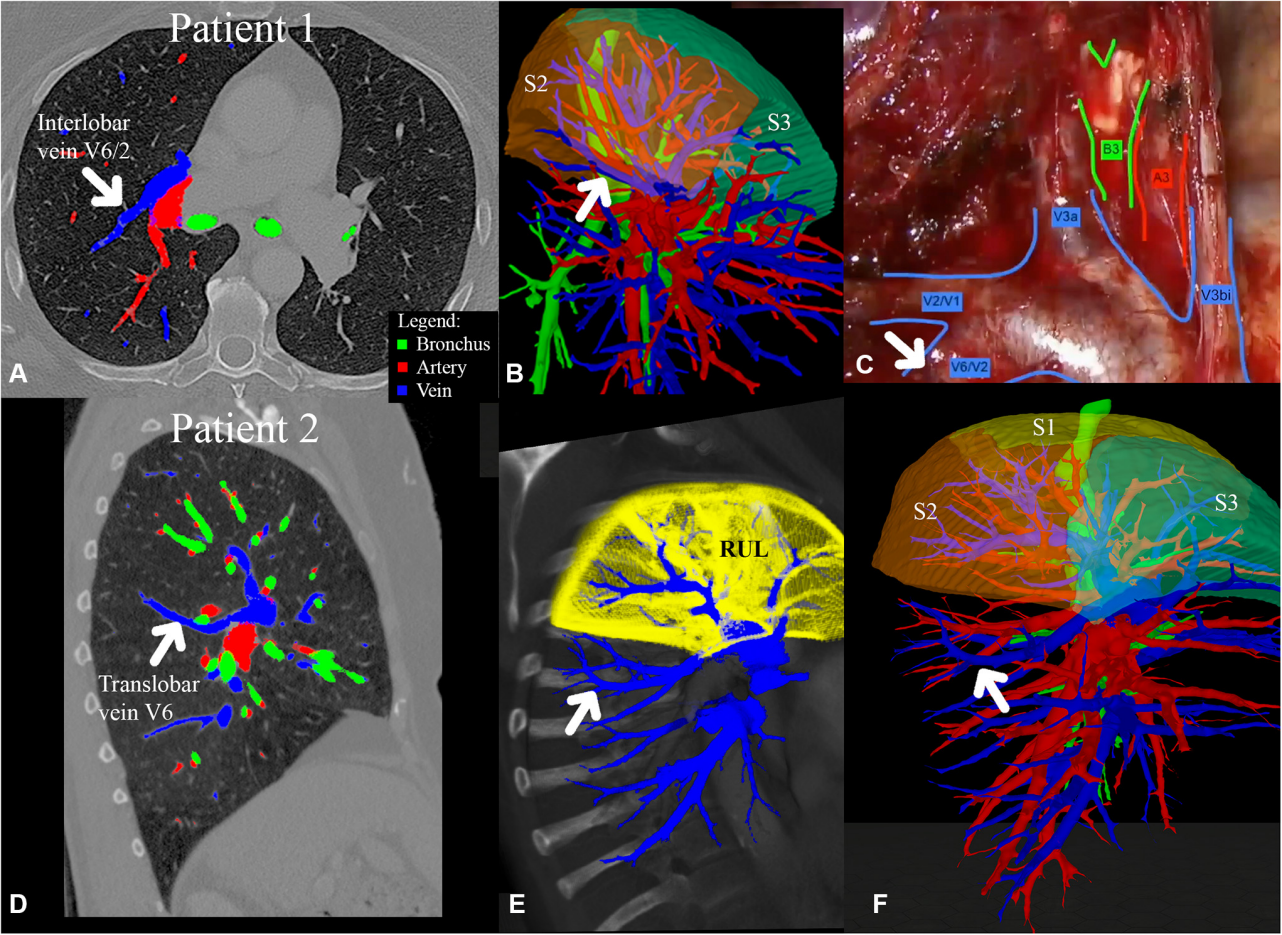
Two-dimensional computed tomography (2D-Ct), virtual reality, and 3-dimensional visualization of cases 1 and 2. A, Case 1: 2D-CT. B, Pulmo3D, lateral view. C, Intraoperative representation of interlobar vein V6/2 (*white arrow* points to interlobar vein V6/2). D, Case 2: 2D-CT. E, PulmoVR lateral view. F, Pulmo3D, lateral view of translobar vein of S6 (*white arrow* points to translobar vein V6). *A*, Artery; *B*, bronchus; *V*, vein; *RUL*, right upper lobe; *S*, segment.

Case 1 was a 70-year-old woman with a histologically proven adenocarcinoma, cT1a N0 M0, in segment 3 of the right upper lobe (RUL). A segment 3 resection was planned. Standard CT scan showed that both fissures were absent in the right lung (Figure 1, *A*). During VR visualization, an interlobar vein between segment 2 and segment 6 draining into the superior pulmonary vein (SPV) was observed (Figure 1, *B*). During surgery, the aberrant drainage was confirmed and the vein was spared, after opening of the horizontal fissure by bluntly dissecting a tunnel between the SPV's and the horizontal fissure (Figure 1, *C*).

Case 2 was a 74-year-old patient with a cT2b N0 M0 adenocarcinoma in the RUL. Standard CT raised the suspicion (due to fissural crossing of a venous structure) of venous drainage of segment 6 into the SPV (Figure 1, *D*), which was confirmed by PulmoVR/Pulmo3D visualization (Figure 1, *E* and *F*). A lobectomy was performed. To spare V6, first, the horizontal fissure was opened with endostaplers similarly to the previous case, by creating a tunnel between the SPV and the horizontal fissure. After that, the RUL veins were ligated selectively and V6 was spared.

**Figure 2:**
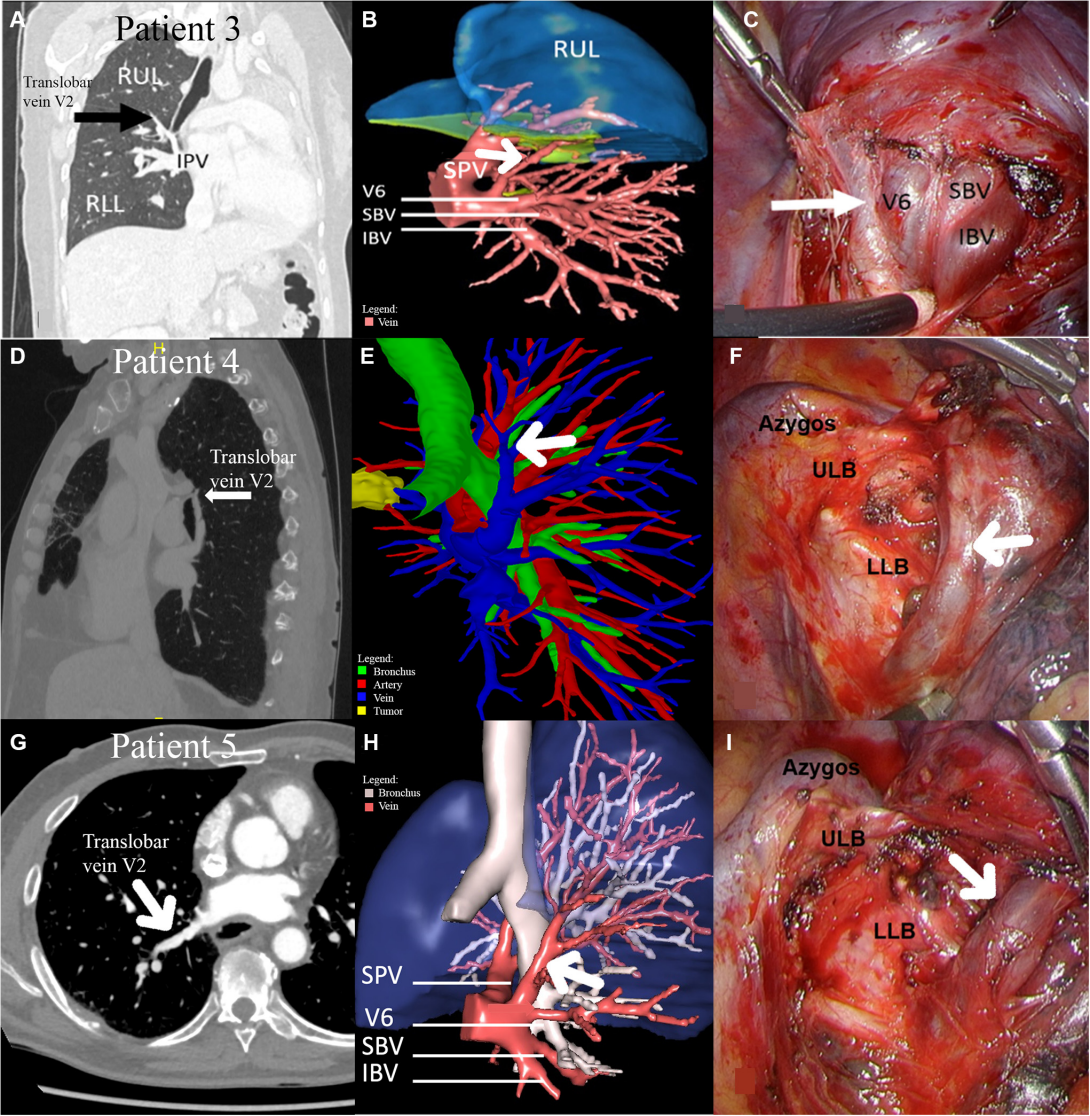
Two-dimensional computed tomography (2D-CT) and 3-dimensional and intraoperative views of retrobronchial veins in cases 3, 4, and 5. Case 3: 2D-CT (A), dorsal view (B), and intraoperative (C) representation of retrobronchial vein of the right upper lobe (RUL) into the inferior pulmonary vein (IPV). Case 4: 2D-CT (D), Pulmo3D, dorsal-medial view (E), and intraoperative (F) view of translobar vein V2. Case 5: 2D-CT (G), dorsal view (H), and intraoperative (I) view of retrobronchial V2 to IPV (*black arrow* (A) and *white arrow* (B-I) indicate translobar vein V2). *V*, Vein; *RUL*, right upper lobe; *IPV*, inferior pulmonary vein; *RLL*, right lower lobe; *SPV*, superior pulmonary vein; *SBV*, superior basal vein; *IBV*, Inferior basal vein; *ULB*, upper lobe bronchus; *LLB*, lower lobe bronchus.

Case 3 was a 30-year-old woman who was referred for a single metastasis in segment 10 of the right lower lobe. A basal segmentectomy was planned. 2D-CT (Figure 2, *A*) and 3D-planning (Figure 2, *B*) demonstrated that segment 2 was mainly drained by a retrobronchial vein, which was the upper tributary of the inferior pulmonary vein (IPV). This vein could be spared by approaching the IPV vein from the dorsal hilum (Figure 2, *C*). After identification and isolation of V2 and V6, the remaining basal veins were ligated.

Case 4 was a 70-year-old patient who was admitted for an adenocarcinoma cT2a N0 M0 located in segment 3 and invading the middle lobe through the fissure. A superior bilobectomy was indicated. 2D-CT suggested a translobar retrobronchial vein of segment 2 (Figure 2, *D*), confirmed by 3D visualization (Figure 2, *E*). During surgery, the vein coming from segment 2 and crossing posteriorly the intermedial bronchus perpendicularly was confirmed (Figure 2, *F*). This vein was also identified by a dorsal approach of the hilum, and subsequently clipped to preserve other IPV tributaries.

Case 5 was a 73-year-old man who presented with a 30-mm ground glass opacity in the right apical segment. Because primary non–small cell lung cancer cT1c N0 M0 tumor was suspected, a segment 1 and 2 segmentectomy was planned. 3D visualization showed a right single (common ostium) pulmonary vein with direct branching of a retrobronchial vein draining segment 2 (Figure 2, *H*). One anteriorly located SPV was draining segment 1 and segment 3. Dorsal approach of the hilum provided a clear view on V2, which could selectively be clipped for the segment 1 and 2 segmentectomy (Figure 2, *I*).

## Conclusions

Over the past few years, an increasing number of more complex sublobar resections have been performed. This raises the need to thoroughly analyze the bronchovascular anatomy pre-operatively to avoid unforeseen circumstances during the procedure. Bronchovascular anatomy can be assessed more easily compared with standard CT based on 3D modeling of patient-specific lung anatomy. In these cases, possible congestion of the venous drainage of the remaining lung lobes/segments could be prevented. In the literature, venous drainage of segment 6 into the SPV, as seen in patients 1 and 2, was observed in approximately 1.5%.^2^ Drainage of segment 2 into the IPV was described in 1.9%.^5^ Single right pulmonary vein drainage is very rare, and incidence is unknown.

Experienced consequences of ligation of translobar venous structures depend on the size of the ligated vein. One of the authors had experienced 1 case of translobar vein ligation, resulting in some venous congestion in the lung parenchym. Patient had no symptoms, but congestion remained visible on follow-up CT scans. On the other hand, we experienced venous congestion after lung transplantation, which did show a lung lobe that resembled decompensation. During reoperation, the lung lobe was hepatized and very fragile. Eventually, this can cause lung (lobe) ischemia if persistent.
